# Plasmatic miR-210, miR-221 and miR-1233 profile: potential liquid biopsies candidates for renal cell carcinoma

**DOI:** 10.18632/oncotarget.21733

**Published:** 2017-10-11

**Authors:** Francisca Dias, Ana Luísa Teixeira, Marta Ferreira, Bárbara Adem, Nuno Bastos, Joana Vieira, Mara Fernandes, Maria Inês Sequeira, Joaquina Maurício, Francisco Lobo, António Morais, Jorge Oliveira, Klaas Kok, Rui Medeiros

**Affiliations:** ^1^ Molecular Oncology and Viral Pathology Group, IPO-Porto Research Center (CI-IPOP), Portuguese Oncology Institute of Porto (IPO-Porto), Porto, Portugal; ^2^ Research Department, LPCC- Portuguese League Against Cancer (NRNorte), Porto, Portugal; ^3^ ICBAS, Abel Salazar Institute for the Biomedical Sciences, University of Porto, Porto, Portugal; ^4^ Medical Oncology Department of the Portuguese Oncology Institute of Porto (IPO-Porto), Porto, Portugal; ^5^ FMUP, Faculty of Medicine, University of Porto, Porto, Portugal; ^6^ Genetics Department of the Portuguese Oncology Institute of Porto (IPO-Porto), Porto, Portugal; ^7^ Urology Department of the Portuguese Oncology Institute of Porto (IPO-Porto), Porto, Portugal; ^8^ Department of Genetics, University Medical Center, Groningen, The Netherlands; ^9^ CEBIMED, Faculty of Health Sciences, Fernando Pessoa University, Porto, Portugal

**Keywords:** circulating miRNAs, renal cell carcinoma, prognostic biomarkers

## Abstract

Renal cell carcinoma (RCC) represents a challenge for clinicians since the nonexistence of screening and monitoring tests contributes to the fact that one-third of patients are diagnosed with metastatic disease and 20–40% of the remaining patients will also develop metastasis. Modern medicine is now trying to establish circulating biomolecules as the gold standard of biomarkers. Among the molecules that can be released from tumor cells we can find microRNAs. The aim of this study was to evaluate the applicability of cancer-related miR-210, miR-218, miR-221 and miR-1233 as prognostic biomarkers for RCC. Patients with higher levels of miR-210, miR-221 and miR-1233 presented a higher risk of specific death by RCC and a lower cancer-specific survival. The addition of miR-210, miR-221 and miR-1233 plasma levels information improved the capacity to predict death by cancer in 8, 4% when compared to the current variables used by clinicians. We also verified that hypoxia stimulates the release of miR-210 and miR-1233 from HKC-8, RCC-FG2 and 786-O cell lines. These results support the addition of circulating microRNAs as prognostic biomarkers for RCC.

## INTRODUCTION

Renal cell carcinoma (RCC) is the most common solid cancer of the adult kidney, accounting for approximately 90% of kidney neoplasms and 3% of all adult malignancies [1]. The most common histological RCC type is the clear cell RCC (ccRCC), which accounts for 80–90% of all RCCs. Worldwide RCC mortality currently exceeds 100.000 patients each year, with the incidence and mortality rates increasing by 2–3% per decade [2]. This reality and the nonexistence of screening and monitoring tests, contributes to the fact that one-third of patients are diagnosed with metastatic disease and 20–40% of the RCC patient’s submitted to nephrectomy will also develop metastasis [3]. Metastatic ccRCC remains incurable, but the prognosis for recurrent ccRCC varies widely and it has been reported that detecting early relapse can improve a patient’s prognosis [4].

The current gold standard of cancer diagnosis is the histological examination of tissue, mainly obtained by biopsy. However this procedure is invasive, expensive and present risks for the patient, which emphasizes the need for alternative diagnostic techniques. Liquid Biopsies hold great clinical promise, as their non-invasive nature allows for rapid, economical and multiple sampling. These features allow their use in screening programs and the close monitoring of disease progression and treatment response, allowing earlier intervention and a dynamic treatment management [5]. Among the possible non-invasive biomarkers that have been studied in RCC, the ones that seem more promising are the microRNAs (miRNAs), since they can be detected using non-invasive procedures and are easier to quantify when compared to other molecules [6]. MiRNAs are small (18–24 nucleotides) non-coding RNAs that are responsible for the regulation of gene expression at a post-transcriptional level and have been widely studied in oncology since they are potent modulators of cellular behaviour and tumoral microenvironment [7, 8]. As a single miRNA may target up to several hundred mRNAs, aberrant miRNA expression may affect a multitude of transcripts and profoundly influence cancer-related signaling pathways [9]. MiRNAs are also present high stability in biofluids since they can be actively secreted from cells inside exosomes and microvesicles or circulate free in complex with proteins such as RNA-binding proteins, lipoproteins, high density lipoproteins (HDLs) and argonoute proteins [10]. The multitude of ways in which miRNAs can be released into circulation gives tumor cells the power to modulate the human body’s response to their own advantage [10]. There is evidence that miRNAs regulate the “hallmarks of cancer”, including the hypoxic microenvironment, a well established cellular characteristic of ccRCC [5]. Hypoxia is a unique environmental stress that induces global changes in a complex regulatory network of transcription factors and signaling pathways in order to coordinate cellular adaptations in metabolism, proliferation, DNA repair, and apoptosis [11]. One of the early molecular events in the oncobiology of ccRCC is the loss of *von Hippel Lindau* (*VHL)* gene which leads to an increase of *Hypoxia Inducible Factor alpha* (HIF-α) and, consequently, triggers an hypoxic response from the cell [12, 13]. Among the miRNAs regulated by hypoxia, we can find miR-210, miR-218 and miR-1233. MiR-210 expression is induced by hypoxia, which makes this miRNA an accurate indicator of the hypoxia state [14]. This miRNA is widely studied in cancer, including RCC, however, studies of its expression in biofluids are few and present contradictory results [15–18]. MiR-218 is considered a tumor suppressor miRNA in RCC and its expression is downregulated by hypoxia [19]. The studies regarding this miRNA were only performed in cell lines and tissue samples, so it would be interesting to evaluate its behavior in patients biofluids [20–23]. HIF1-α induces the transcription of multiple proangiogenic and growth factors including the vascular endothelial growth factor (VEGF) that subsequently activates a number of downstream pathways by binding mainly to VEGFR-2 [24]. MiR-221 targets VEGFR-2, its involved in the EGFR pathway activation and its overexpression in plasma samples was associated with a lower progression free survival (PFS) and lower overall survival (OS) in RCC patients by our group [25, 26]. MiR-1233 is considered an oncomiRNA since it targets p53, inhibiting its function in RCC [18]. However, so far there is only one study regarding miR-1233 expression in in RCC and, given the importance of the relation of p53 with HIF, it is important to further study the impact of this miRNA in RCC progression.

Regarding circulating miRNAs in RCC, only a few have been suggested as potential biomarkers for diagnosis and/or prognosis [6, 25]. Despite promising, the miRNAs were studied in small cohorts and few were replicated by other groups, which empathizes the need for more studies in order to replicate, validate and establish circulating miRNAs as RCC biomarkers [6]. Our aim in this study is the evaluation of the impact of plasma levels of miR-210, miR-218, miR-221 and miR-1233 in clinical endpoints and their association with clinicopathological characteristics of RCC patients.

## RESULTS

### MiR-210, miR-218, miR-221 and miR-1233 are released from tumor cell lines

**Figure 1:**
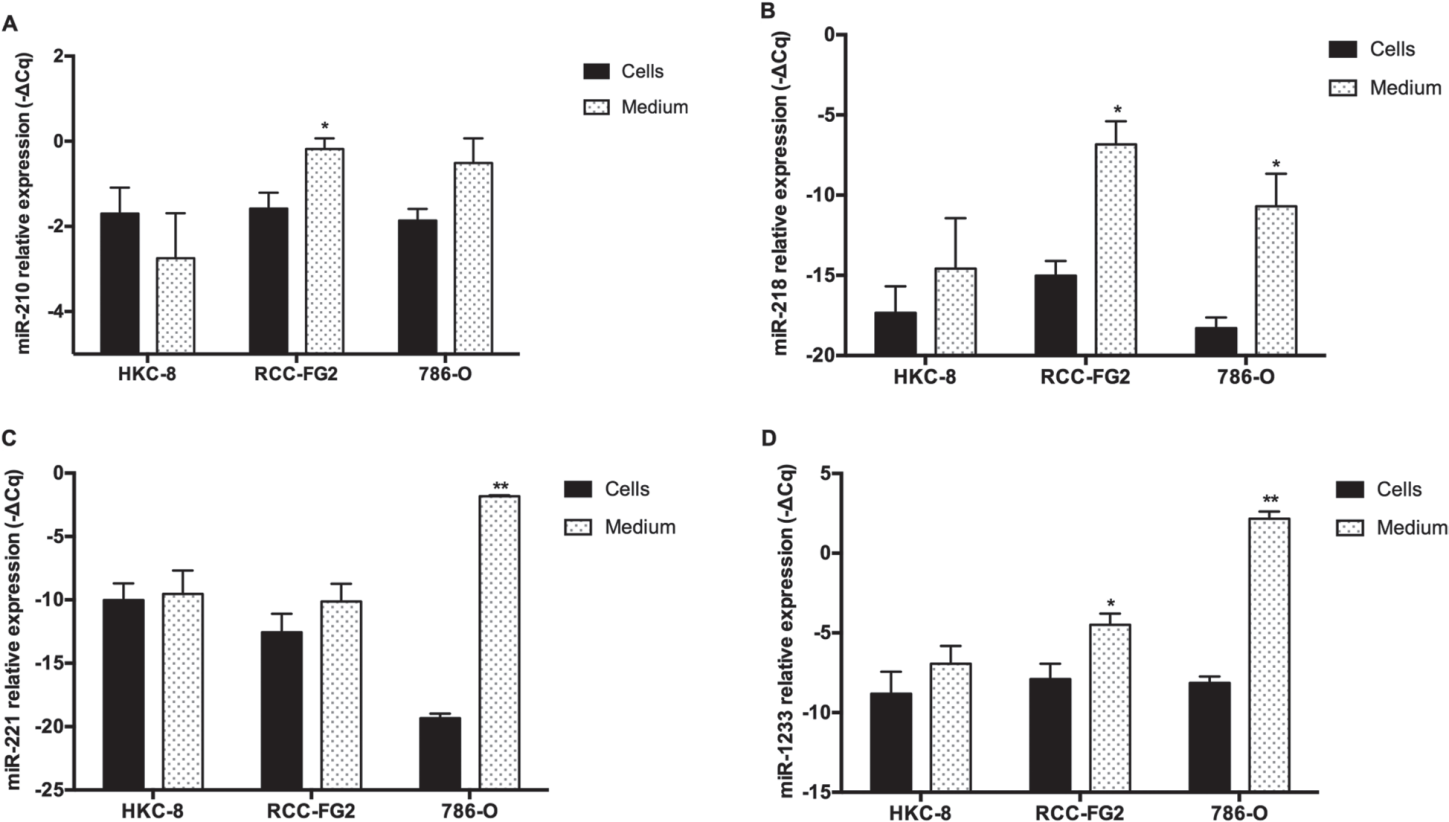
Intracellular and extracellular expression of miR-210, miR-218, miR-221 miR-1233 in HKC-8, 786-O and RCC-FG2 cell lines The bars represent the –ΔCq of the miRNAs plasmatic expression normalized to RNU48. (**A**) miR-210 levels in HKC-8, RCC-FG2 and 786-O cell lines and respective medium; (**B**) miR-218 levels in HKC-8, RCC-FG2 and 786-O cell lines and respective medium; (**C**) miR-221 levels in HKC-8, RCC-FG2 and 786-O cell lines and respective medium and (**D**) miR-1233 levels in HKC-8, RCC-FG2 and 786-O cell lines and respective medium. (Mean ± Std.Error; ^*^P < 0.05, ^**^P < 0.001).

To validate the hypothesis that RCC cells may excrete these miRNAs into circulation, we performed an *in vitro* study in which we evaluate the levels of miR-210, miR-218, miR-221 and miR-1233, intracellularly and in the culture medium, of HKC-8, RCC-FG2 and 786-O cell lines. According to our results, with exception of miR-221 (Fold-change 5, *P* = 0.260), all the miRNAs are excreted from RCC-FG2 cells into their culture medium (Fold-change: miR-210: 3, *P* = 0.020; miR-218: 302, *P* = 0.002 and miR-1233: 11, *P* = 0.021). Regarding the 786-O cells, all the miRNAs were also excreted to the medium, with exception of miR-210 that only showed a tendency for excretion to medium (Fold-change: miR-210: 3, *P* = 0.058; miR-218: 193, *P* = 0.017, miR-221: 853 363, *P* < 0.001 and miR-1233: 1260, *P* < 0.001). Finally, none of the four miRNAs were excreted from HKC-8 cells (Fold-change: miR-210: 0.5, *P* = 0.744; miR-218: 7, *P* = 0.482; miR-221: 1.4, *P* = 0.838 and miR-1233: 4, *P* = 0.327) (Figure 1).

### Plasma levels of miR-210, miR-218, and miR-1233 in RCC patients and their association with clinicopathologic characteristics

**Figure 2:**
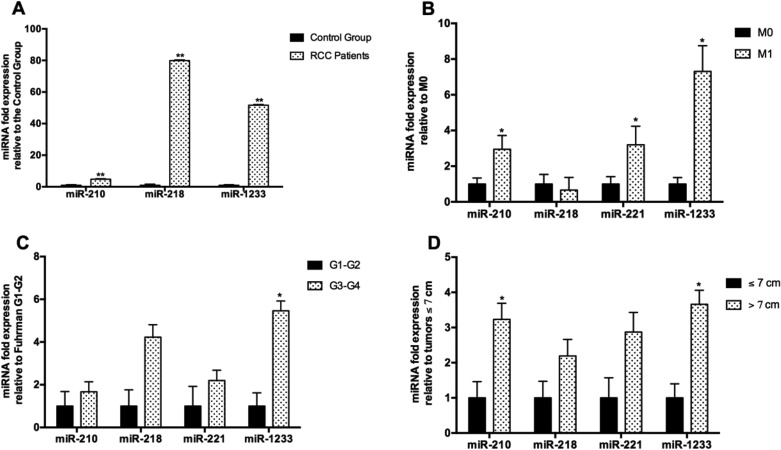
Fold-change of the plasmatic levels of miR-210, miR-218 and miR-1233 according to clinicopathological characteristics The bars represent the 2^-ΔΔCq^ as a fold-change in miRNA plasmatic expression normalized to RNU48. Expression levels shown are means of three technical replicates for each sample. (**A**) RCC patients *vs* healthy individuals; (**B**) Tumor ≤7 cm *vs* tumors > 7 cm); (**C**) Fuhrman grade G1-G2 *vs* Fuhrman grade G3-G4); and (**D**) No presence of metastasis ate the time of diagnosis (M0) vs Presence of metastasis at the time of diagnosis (M1). (Mean ± Std.Error; ^*^P < 0.05, ^**^P < 0.001).

According to our results, we observed a significant increase in the plasma levels of miR-210, miR-218 and miR-1233 in RCC patients, when compared to healthy individuals (Fold-change: miR-210: 5, *P* < 0.001; miR-218: 80, *P* < 0.001; and miR-1233: 52, *P* < 0.001) (Figure 2A). We did not performed this analysis for miR-221 because it was already made by our group in a previous study, were we concluded that RCC patients presented higher plasma levels of miR-221 [25].

However, we did not found any statistical differences between miR-210, miR-218 and miR-1233 levels according to gender (miR-210: *P* = 0.626, miR-218: *P* = 0.577 and miR-1233: *P* = 0.822), tumor subtype (miR-210: *P* = 0.138, miR-218: *P* = 0.160, miR-1233: *P* = 0.132) and age (miR-210: *P* = 0.527, miR-218: *P* = 0.413, miR-1233: *P* = 0.377) in the patients group.

Regarding the clinicopathologic characteristics, we observed that patients with tumors larger than 7 cm present higher levels of miR-210 and miR-1233 (Fold-change: miR-210: 3, *P* = 0.022; miR-218: 2, *P* = 0.249; miR-221: 3, *P* = 0.066 and miR-1233: 4, *P* = 0.003) (Figure 2D). When we compare the miRNAs expression levels with the Fuhrman nuclear grade, only miR-1233 was associated with higher Fuhrman grades (Fold-change: miR-210: 2, *P* = 0.381; miR-218: 4, *P* = 0.051; miR-221: 2, *P* = 0.246 and miR-1233: 5 *P* = 0.004) (Figure 2C). We also observed that higher levels of miR-210 and miR-1233 were associated with presence of metastasis at the time of diagnosis when compared with patients with localized disease (Fold-change: miR-210: 3, *P* = 0.045; miR-218: 0.7, *P* = 0.579; miR-221: 3, *P* = 0.030 and miR-1233 and miR-1233: 7, *P* = 0.029) (Figure 2B).

### Higher plasmatic levels of miR-210, miR-221 and miR-1233 and cancer-specific survival

**Figure 3:**
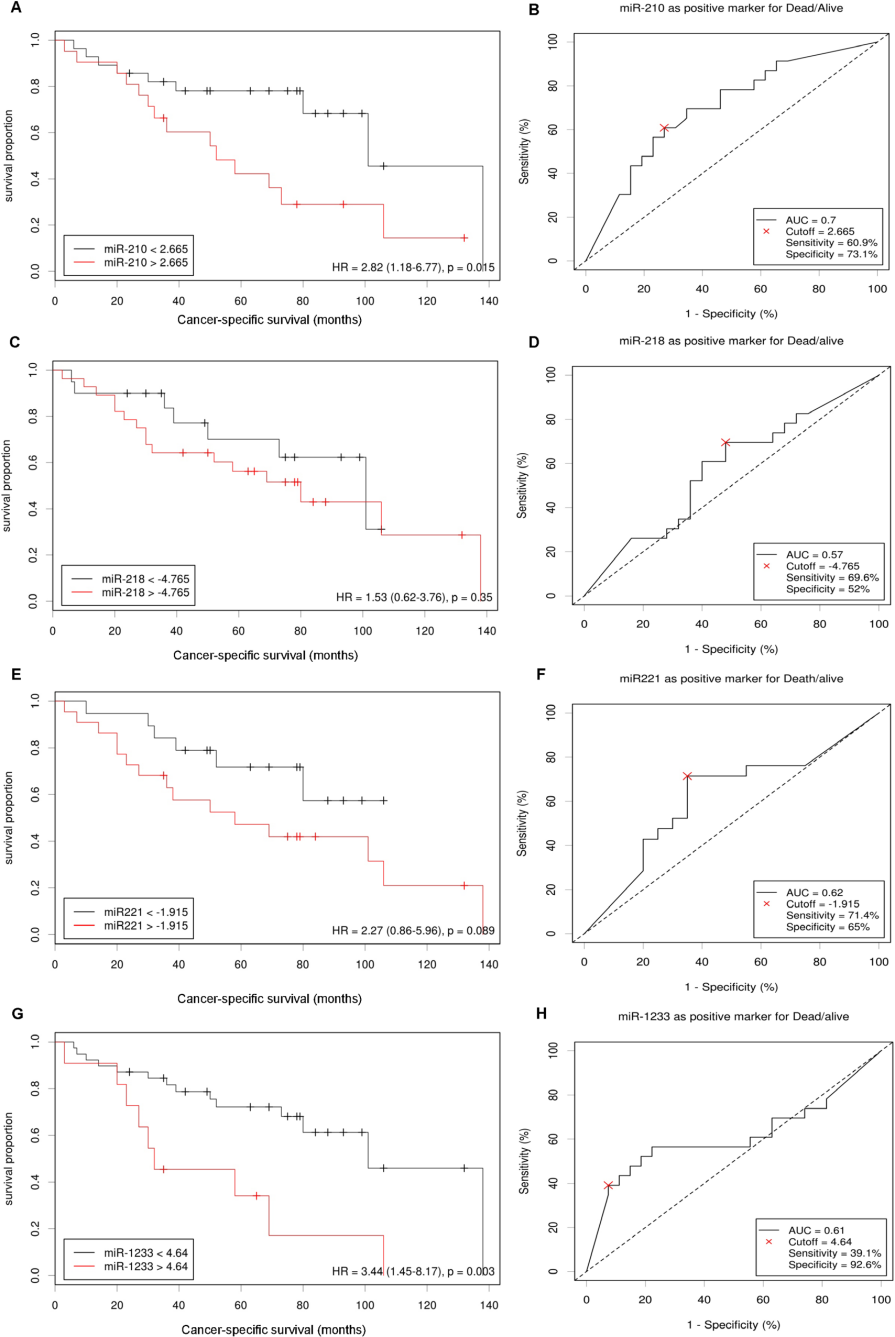
miR-210, miR-218, miR-221 and miR-1233 prognostic roles in patients with RCC Cut-offs for “high” and “low” expression of miR-210 (**A**), miR-218 (**C**), miR-221 (**E**) and miR-1233 (**G**) were identified by the online web application *Cutoff Finder*. The optimal cut-off is defined as the point with the most significant (log-rank test) split and the effect of each miRNA in cancer-specific survival is presented in the corresponding Kaplan-Meier plot. The *Cutoff Finder* also allowed to assess the quality of the prediction through the construction of ROC curves using the optimal cut-off points established for miR-210 (**B**), miR-218 (**D**), miR-221 (**F**) and miR-1233 (**H**).

**Figure 4:**
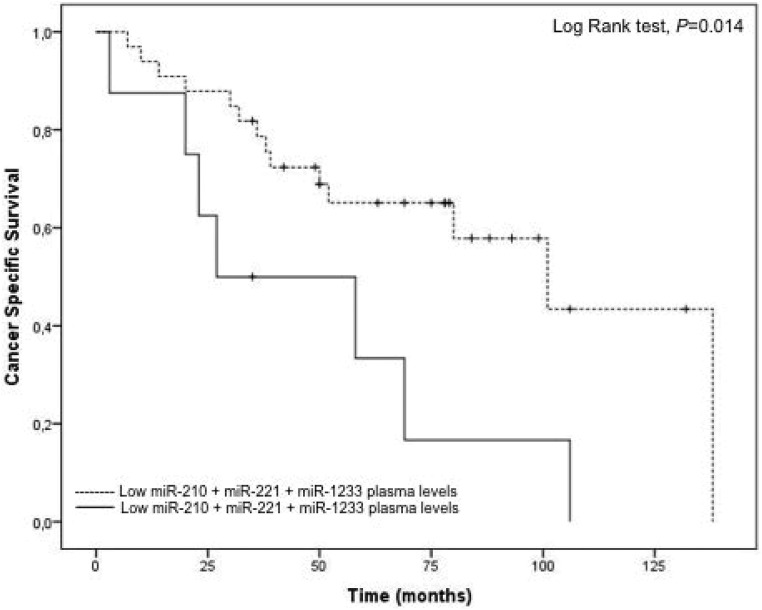
Cancer-specific survival according to combined expression of miR-210, miR-221 and miR-1233 plasma levels in RCC patients Patients with higher expression of miR-210, miR-221 and miR-1233 combined present a lower cancer-specific survival (Log Rank test, *P* = 0.014).

The fifty patients in the entire cohort were separated into miR-210, miR-218, miR-221 and miR-1233 high risk (higher plasma levels) and low risk (lower plasma levels) groups using *Cutoff Finder* software (http://molpath.charite.de/cutoff), to generate the optimum cut-off score for their normalized plasma expression (-ΔCq). The Kaplan-Meier analysis showed that patients with higher levels of miR-210 and miR-1233 (high risk groups) present a significantly lower cancer-specific survival (*P* = 0.015, *P* = 0.003, respectively) (Figure 3, left panel). MiR-218 plasma levels weren’t associated with cancer-specific survival (*P* = 0.350) and miR-221 showed a tendency to be associated with cancer-specific survival (*P* = 0.089) Additionally, the *Cutoff Finder* software also allowed us to apply a ROC analysis using the optimum cut-off score generated, which can be observed in Figure 3B, 3D, 3F and 3H (right panel). Regarding miR-210, the sensitivity was 60.9% and the specificity was 73.1% (AUC = 0.70); for miR-221, the sensitivity was 71.4% and the specificity was 65% (AUC = 0.62) and for miR-1233 the sensitivity was 39.1% and the specificity 92.6% (AUC 0.61). We also observed an addictive effect of the combination of the plasma levels of miR-210, miR-221 and miR-1233. In fact, the cancer-specific survival was significantly lower in patients’ with higher levels of miR-210, miR-221 and miR-1233 (48 *versus* 91 months) (Figure 4). Furthermore, multivariate Cox regression model using tumor TNM stage (I /II versus III/IV), Fuhrman nuclear grade (G1/G2 versus G3/G4), Age (> 60 years) and gender as co-variants, demonstrated a higher risk of specific death by RCC in patients who presented simustaneously higher levels of miR-210, mir-221 and miR-1233 (HR = 3.02, 95%CI 1.19–7.64, *P* = 0.014). The concordance (*c*) index was used to compare the predictive ability of different prognostic variables associated with RCC overall survival; the predictive value was assessed with Harrell’s concordance indexes, where a c index of 1 indicates perfect concordance [27]. Tumor TNM stage and Fuhrman nuclear grade are well-known prognostic factors for cancer progression. In our study, the predictive value of tumour TNM stage (I/II versus III/IV), Fuhrman nuclear grade (G1/G2 versus G3/G4), and age (> 60 years) for poor cancer-specific survival (death by RCC) was 0.744 (model 1). However, this predictive ability increased to 0.828 (model 2) with the addition of the information regarding miR-210, miR-221 and miR-1233 plasma levels. The addition of miR-210, miR-221 and miR-1233 plasma levels information improved the capacity to predict death by cancer in 8.4% compared with model 1 (Table 1).

**Table 1:** Predictive models of death by RCC according to different prognostic factors

	HR	95% CI	*P* value	*c* index
**Model 1**				
Tumor TNM stage (I and II vs III and IV), Fuhrman nuclear grade (G1 and G2 vs G3 and G4), Age (> 60 years) and Gender	3.90	1.76–8.64	< 0.001	0.744
**Model 2**				
miR-210 + miR-221 + miR-1233 plasma expression, Tumor TNM stage (I and II vs III and IV), Fuhrman nuclear grade (G1 and G2 vs G3 and G4), Age (> 60 years) and Gender	3.89	1.26–12.01	0.018	0.828

### Accute hypoxia exposure stimulates the release of miR-210 and miR-1233 from normal and tumor cell lines and interferes with CXCR4 mRNA expression

**Figure 5:**
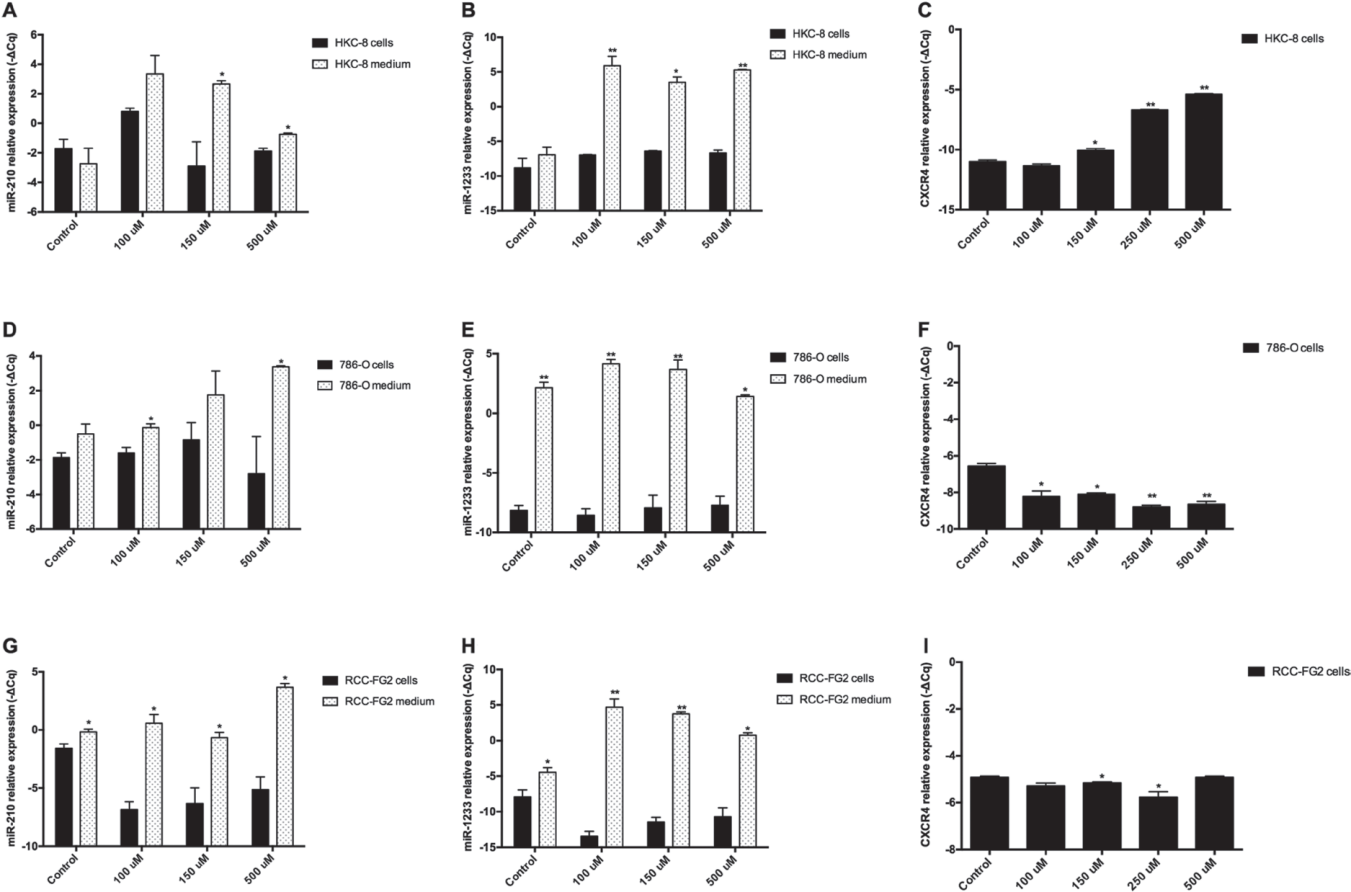
Extracellular expression of miR-210, miR-1233 and CXCR4 in HKC-8, 786-O and RCC-FG2 cell lines after hypoxia induction The bars represent the –ΔCq of miR-210, miR-1233 and CXC4 after hypoxia stimulation with crescent doses of CoCl_2_ for each cell line. (**A**) miR-210 levels in HKC-8 cell line after CoCl_2_ stimulation; (**B**) miR-1233 levels in HKC-8 cell line after CoCl_2_ stimulation; (**C**) CXCR4 levels in HKC-8 cell line after CoCl_2_ stimulation; (**D**) miR-210 levels in 786-O cell line after CoCl_2_ stimulation; (**E**) miR-1233 levels in 786-O cell line after CoCl_2_ stimulation; (**F**) CXCR4 levels in786-O cell line after CoCl_2_ stimulation; (**G**) miR-210 levels in RCC-FG2 cell line after CoCl_2_ stimulation; (**H**) miR-1233 levels in RCC-FG2 cell line after CoCl_2_ stimulation; (**I**) CXCR4 levels in RCC-FG2 cell line after CoCl_2_ stimulation; The –ΔCq of miR-210 and miR-1233 was normalized to RNU48 and the CXCR4 –ΔCq was normalized to GUSB. (Mean ± Std.Error; ^*^P < 0.05, ^**^P < 0.001).

In an attempt to validate the hypothesis that the hypoxia is involved in the release of these miRNAs into circulation, we stimulated all the cell lines with crescent doses of cobalt chloride (CoCl_2_), a well known hypoxia inducer, during 24h and evaluated its effects on miR-210 and miR-1233 excretion in all cell lines (Figure 5). In normal conditions, the HKC-8 cell line doesn’t excrete none of the miRNAs to the cell medium. However, when we stimulate the cells with CoCl_2_, they start to excrete miR-210 and miR-1233 (Figure 5A–5C). Interestingly, when we do the same stimulus in the RCC cell lines, we also verify an increase of the miR-210 and miR-1233 excretion but it’s not as accentuated as for the HKC-8 cell line (Figure 5D, 5E, 5G and 5H).

Additionally, we also measured the mRNA expression of CXCR4, a well known molecule involved in cancer progression and metastasis and what we observed was that, with the acute hypoxic stimulus, the HKC8 cell line started to express more CXCR4 mRNA and that expression was dependent of the CoCl_2_ concentration (Figure 5C). However, in the 786-O cell line the CXCR4 mRNA expression diminished with the increase of CoCl_2_ concentration and in the FG-2 cell line the CXCR4 mRNA expression was approximately the same despite the increase of CoCl_2_ concentration (Figure 5F and 5I).

## DISCUSSION

Currently, no standard approaches to biomarker sampling or analysis have been adopted for RCC since many of the potential tumor markers are still under active investigation for further validation [28]. There are several factors that must be considered when choosing miRNAs as candidate prognostic biomarkers for RCC. First, the fold-change of the miRNA should be significant enough to discriminate RCC patients from healthy individuals. Second, the biological function and carcinogenesis mechanism of each miRNA should be thoroughly investigated in RCC since a better understanding of the targeted genes of the miRNAs would advance their use in clinical settings. Last but not least, rigorous validation and demonstration of reproducibility in independent cohorts of patients are necessary to confirm the prognostic value of miRNAs [29].

Some authors state that the most miRNA enriched biofluid is the plasma and inclusively there is a study showing the superiority of plasma over serum for circulating miRNAs analysis [30]. This study is based on the release of platelets or white blood cells miRNA contents to the serum during the coagulation process, which compromise the miRNA contend in serum [30, 31]. The existing studies in circulating samples that evaluated miR-210 and miR-1233 were made in serum samples and miR-218 was never studied in biofluids [15–18]. Only miR-221 was characterized in plasma samples of RCC patients, in previous studies from our group [25, 32]. To the best of our knowledge, this is the first study that evaluates the plasma levels of miR-210, miR-218 and miR-1233 in RCC.

Our *in vitro* study demonstrated that RCC-FG2 cell line excreted miR-210, miR-218 and miR-1233 to the extracellular medium and that 786-O cell line excreted miR-218, miR-221 and miR-1233 to the medium and also a tendency to excrete miR-210. Those results were validated *in vivo* when we compared plasma samples from RCC patients with healthy controls. Additionally we observed that patients with tumors higher than 7 cm and patients that presented metastasis at the time of diagnosis presented higher levels of miR-210 and miR-1233, suggesting that these two miRNAs are related to tumor aggressiveness alongside miR-221 that we previously reported as having higher plasma concentrations in RCC patients [25]. Additionally, miR-1233 was the only miRNA associated with higher Fuhrman grades. Although we didn’t find any association between miR-218 and the clinicalphatologic features, our results regarding this miRNA are interesting because it is secreted by the tumor cell line and its also higher in the plasma of the RCC patients. This suggests that miR-218 is indeed secreted by the tumor, but probably with a different purpose. Since miR-218 is described in the literature as a tumor suppressor miRNA and is down-regulated in RCC tumor samples, we hypothesize that the excretion of miR-218 is a mechanism that tumor cells use in order to prevent its tumor suppression activity [21, 22].

When we analyzed the individual impact of the expression of miR-210 and miR-1233 in clinical endpoints, we observed that they were both associated with a lower cancer-specific survival, while miR-221 only showed a tendency towards that. Subsequently, we analyzed the impact of higher levels of the three miRNAs and we observed that patients presenting higher levels of miR-210, miR-221 and miR-1233 combined presented a higher risk of specific death by RCC and a lower cancer-specific survival. Additionally we created a model of death prediction by RCC using the standard variables used in the clinic and compared it to a model were we added the expression profile of miR-210, miR-221 and miR-1233 and we observed that the addiction of the plasma levels of miR-210, miR-221 and miR-1233 improved the capacity to predict death by RCC in when compared to the first model. These results suggest that miR-210, miR-221 and miR-1233 combined are potential prognostic profile of biomarkers for RCC and also open the door for the addition of microRNAs in predictive models of death by RCC.

Additionally, we also evaluated the impact of acute hypoxia in the two miRNAs that presented a significant individual impact in cancer-specific survival: miR-210 and miR-1233. After the addition of crescent concentrations of CoCl_2_ to all the cell lines we observed that indeed hypoxia was part of the process by which cells excrete miRNAs into the cell medium. That effect is very marked in the HKC-8 cell line, were we observed that, in normal conditions, this cell line doesn’t excretes neither miR-210 nor miR-1233 but when we stimulate then with crescent doses of hypoxia both miRNAs are excreted to the cell medium. The crescent hypoxia is also associated with crescent production of CXCR4 mRNA, a potent angiogenic and tumor progression inducer. In the ccRCC cell lines (RCC-FG2 and 786-O) we also observe that the hypoxic stimulus also increases the release of miR-210 and miR-1233 but not as markedly as in the HKC-8 cell line, which may be due to the fact that both these cell lines already present a basal excretion of theses miRNAs due to their malignancy and RCC phenotype. Interestingly, the hypoxia stiumulus as the opposite effect in 786-O and RCC-FG2 cell lines when compared to the HKC-8 cell line, which led us to the conclusion that CXCR4 may only act in the first stages of hypoxia.

In conclusion, the stimulus to hypoxia, which translates in a higher grade of cell proliferation, angiogenesis and metastatic potential, in patients with higher levels of miR-210 and miR-1233 is associated with a lower cancer specific survival, resulting in a higher risk of death by RCC. For the first time it was demonstrated that the evaluation of the combined miR-210, miR-221 and miR-1233 profile could allow a better monitorization of RCC patients’ by distinguishing poor from favorable risk patients. In the future it would be interesting to improve our RCC death prediction model by studying and adding more plasma miRNAs and also to perform more functional studies to better understand the effects of hypoxia on miRNA secretion from the cells. It would also be interesting to study the mechanism by which the tumor cells excreted these miRNAs since recent studies demonstrated that tumor cells use exosomes (small vesicles) to shuttle molecules from the tumor to other locations.

## MATERIALS AND METHODS

### Cell line characterization

Three renal cell lines were used: HKC-8, RCC-FG2 and 786-O. The HKC-8 cell line is an immortalized proximal tubular epithelial renal cell line (PTEC), the RCC-FG2 is a metastatic ccRCC cell line and 786-O is a ccRCC cell line. Both HKC-8 and RCC-FG2 were kindly provided by Dr Klaas Kok (Groningem University, Netherlands) and 786-O was kindly provided by Professor Carmen Jerónimo (IPO-Porto Research Center, Portugal) [33].

### Cell culture

Initially a cryopreserved vial of each cell line was thawed. The RCC-FG2 and 786-O cell lines were maintained in RPMI 1640 (1X) medium (Gibco^®^), supplemented with 10% of FBS (Fetal Bovine Serum) (Gibco^®^) and 1% of Pen-Strep (Gibco^®^). The HKC-8 cell line was kept in DMEM/F12 medium (Gibco^®^), supplemented with ITS (Insuline-transferrine-selenium) (Sigma-Aldrich^®^), Pen-Strep (Gibco^®^), EGF (Epidermal Growth Factor) (*S*igma-Aldrich^®^), Hepes buffer (Gibco^®^) and Hydrocortisone (Sigma-Aldrich^®^). Both cell lines were maintained in a 5% CO_2_ incubator at 37°C.

When the desired confluence was achieved (80–90%) the medium, in which the cells were being cultured, was collected for miRNA extraction and the cells were trypsinized, using 0.05 % trypsin-EDTA (1×) (Gibco^®^) and counted using a Neubauer chamber and Tripan-Blue dye (Gibco^®^). After counting, approximately two million cells were centrifuged to form a pellet for miRNA extraction and the remaining cells were kept in culture. This procedure was repeated five times for each cell line.

### Hypoxia induction

All the cell lines were harvested during the logarithmic period and counted by NanoTech cell countes. Cell suspencions, approximately 200 000 cells/well were seeded in 6-well plates (marca) and cultured in a humidified incubator at 37°C and 5% CO2 for 24 h. Then, CoCl_2_ (Ref C8661, *S*igma-Aldrich^®^), was added in crescent concentrations was added to each well, making final concentrations of 100 μM, 150 μM and 500 μM. The cells were incubated with CoCl_2_ during 24h and after that period the miRNAs were extracted from the cells and respective medium and quantified using the procedures previously described. This experimente was performed two times and in duplicate for each cell line.

### Study population

The validation of the circulating miRNA expression profile was made through a hospital-based study, involving a total of 104 individuals: 54 RCC patients and 50 healthy individuals. All RCC patients were Caucasian from the north of Portugal, with histopathologic diagnosis of RCC, admitted and treated at the Portuguese Oncology Institute of Porto (IPO-Porto) between 1 of September 2003 and 30 of October 2013. The mean age was 60.3 ± 12.1 years, from which 74.1% (*n* = 40) were male and 25.9% (*n* = 14) female. Patients’ clinical characteristics were obtained from their medical records. Tumor classification and staging were established according to the tumor-node-metastasis (TNM) classification system of the American Joint Committee on Cancer (AJCC) 2010, 7^a^ edition (Table 2). For the control group, 50 healthy Caucasian individuals, from which 30% were male (*n* = 16) and 68% (*n* = 34) were female, with no history of cancer, were randomly recruited from the north of Portugal, with a mean age of 43.0 ± 15.5 years.

**Table 2:** Distribution of the clinicopathological factors of the study population

	Cases (*n* = 54)		Control Group (*n* = 50 )	
	*n*	%	*n*	%
Gender				
Male	40	74.1	16	32
Female	14	25.9	34	68
Age				
Mean ± SD	60.3 ± 12.1		43.0 ± 15.5	
Histology				
Clear Cell	39	72.2		
Others	15	27.8		
TNM Stage				
I–II	19	38		
III–IV	31	62		
T				
T1	18	33.4		
T2	5	9.30		
T3	26	48.2		
T4	5	9.30		
N				
N0	6	11.1		
N1	2	3.70		
N2	4	7.40		
Nx	42	77.8		
M				
M0	42	77.8		
M1	12	22.2		
Fuhrman Grade				
G1	1	1.90		
G2	15	27.8		
G3	16	29.6		
G4	19	35.2		
Unknown	3	5.60		

### Sample collection and miRNA and mRNA extraction/ purification

Approximately 8 mL of peripheral blood were collected from all individuals through a standard method of intravenous collection using EDTA tubes. The blood tubes were centrifuged 5 minutes at 3000 rpm at room temperature, in order to separate the plasma fraction from the blood cells.

The miRNA isolation protocol was the same for cultured cells, cultured cells medium and plasma samples. We added an acid phenol-chloroform (5:1) solution (Ambion^®^) to the samples, which, after centrifugation at 15.000 rpm at 5°C for 15 min, allowed the separation of the RNA/microRNA phase. MicroRNA purification was performed using the GRS microRNA kit (Grisp^®^), with adjustments in the manufactured protocol.

The mRNA isolation and purification of the culture cells and cultured cell medium was performed using the GRS Total Blood & Cultures Cells kit *(*Grisp^®^*)*

After isolation, RNA concentration and purity were measured at 260 and 280 nm using the *NanoDrop*^®^ *ND-1000* spectrophotometer.

### cDNA synthesis

The miRNA samples were used as templates for cDNA synthesis using a Taqman^®^MicroRNA *Reverse* Transcription kit (Applied Biosystems^®^) and sequence-specific stem-loop primers for hsa-miR-210-3p, hsa-miR-218-1-3p, hsa-miR-221, hsa-miR-1233-3p and RNU-48. We used RNU-48 as an endogenous control for data normalization since it presented a stable expression pattern among samples and was previously used for data normalization in RCC studies [34]. After protocol optimization the thermal conditions were as follows: 16°C for 30 minutes, followed by 42°C for 60 minutes and 85°C for 10 minutes.

The mRNA samples served as templates for cDNA synthesis using a High Capacity cDNA Reverse Transcription Kit (Applied Biosystems^®^). The thermal conditions for PCR amplification were optimized to fby 37°C for 120 min and 85°C for 5 min for mRNA.

### Real-time PCR relative quantification

The miRNA and mRNA expression levels were analyzed by quantitative real-time PCR. The reactions were carried out on a StepOne™qPCR Real-Time PCR machine, containing 1X Master mix (Applied Biosystems^®^), with 1X probes (TaqMan^®^ microRNA Expression Assays: hsa-miR-210-3p: TM000512, hsa-miR-218-1-3p: TM-002094, hsa-miR-221: TM-002096, hsa-miR-1233-3p: TM-002768 and TaqMan^®^ mRNA Expression Assays: CXCR4: Hs00607978_s1, Applied Biosystems^®^ ), cDNA sample (≈ 50 ng), RNU-48 endogenous control for miRNA normalization (TaqMan^®^ Gene Expression Assays, TM-001006, Applied Biosystems^®^) and Human GUSB (Beta Glucoronidase) endogenous control (Applied Biosystems^®^) for mRNA normalization.

The amplification conditions were as follows: holding stage 95°C for 20 seconds, followed by 45 cycles of 95°C for 1 second and 60°C for 20 seconds. Three technical replicates were made for each sample.

Data analysis was made using StepOne™Sofware v2.2 (Applied Biosystems^®^) with the same baseline and threshold set for each plate, in order to generate quantification cycle (Cq) values for all the miRNAs in each sample.

### Statistical analysis

Statistical analysis was also made using IBM^®^SPSS^®^Statistics software for Windows (Version 22.0). The 2^-ΔΔCq^ method, along with the Student t’ test were used in order to evaluate any statistical differences in the normalized expression levels of the miRNAs here explored. We used Cutoff Finder web application to generate optimum cut-off point for the three deregulated miRNAs in the plasma samples of RCC patients. Cutoff Finder selects the optimum cut-off point, separating patients into high-risk and low-risk groups, by fitting Cox proportional hazard models to the dichotomized variable and the survival variable. The optimal cut-off is defined as the point with the most significant (log-rank test) slip [35]. A Cox porportional hazard model was used to analyze the patients’ cancer-specific survival, considering as covariants TNM stage, Fuhrman nuclear grade and age (> 60 years). The concordance (χ) was used to compare the predictive ability of the association of well-known prognostic variables with the combined plasma levels of miR-210, miR-221 and miR-1233 with χ > 0.5 being considered with a good prediction ability [27].

## Abbreviations

RCC: Renal Cell Carcinoma
ccRCC: clear cell Renal Cell Carcinoma
miRNAs: microRNAs
HDL: high density lipoprotein
HIF-1α: Hypoxia Inducible Factor alpha
PTEC: proximal tubular renal cell line
FISH: Fluorescence *in situ* Hybridization
TNM: tumor-node-metastasis
AJCC: American Joint Committee on Cancer
EFNA3: ephrin A3
PTP1B: tyrosine phosphatase non-receptor type 1
CASP8AP2: caspase 8 associated protein 2
E2F3: E2F transcription factor 3
MNT: MAX network transcriptional repressor
FGFR1: fibroblast growth factor receptor 1
EGFR: Epidermal Growth Factor Receptor
*VHL*: von Hippel Lindau tumor suppressor
*HRAS*: HRas proto-oncogene
*RASSF7*: Ras association domain family member 7
*RAD52*: RAD52 homolog, DNA repair protein
*BLCAP*: bladder cancer associated protein
